# Association of MASLD with Baseline and New-Onset Liver Function Test Elevation in Medical ICU Patients

**DOI:** 10.3390/medicina61122092

**Published:** 2025-11-24

**Authors:** Ali Karataş, Kamil İnci, Nazlıhan Boyacı Dündar, Gülbin Aygencel, Melda Türkoğlu, Ali Osman Taş, Beril Avcı, Cansu Gedik, Mehmet Cindoruk

**Affiliations:** 1 Department of Gastroenterology, Faculty of Medicine, Gazi University, Ankara 06560, Turkey; berilavci91@gmail.com (B.A.); drmehmetcindoruk@gmail.com (M.C.); 2 Department of Intensive Care Unit, Gazi University, Ankara 06560, Turkey; kamilinci@gmail.com (K.İ.); nazlihan_boyaci@yahoo.com (N.B.D.); aygencel@hotmail.com (G.A.); meldaturkoglu@yahoo.com.tr (M.T.); 3 Department of Internal Medicine, Faculty of Medicine, Gazi University, Ankara 06560, Turkey; aosman155@gmail.com (A.O.T.); cansu_gedik98@hotmail.com (C.G.)

**Keywords:** metabolic dysfunction-associated steatotic liver disease, intensive care unit, liver function tests

## Abstract

*Background and Objectives*: Metabolic dysfunction-associated steatotic liver disease (MASLD) is highly prevalent and may influence the outcome of critical illness. Although abnormal liver function tests (LFTs) are frequent in the intensive care unit (ICU), the contribution of MASLD to organ-specific hepatic vulnerability and mortality remains unclear. This study aimed to evaluate whether pre-existing metabolic dysfunction-associated steatotic liver disease (MASLD) is associated with baseline and new-onset liver function test (LFT) abnormalities and with intensive care unit (ICU) outcomes in non-cirrhotic medical ICU patients. *Materials and Methods*: We conducted a retrospective cohort study of adult non-cirrhotic patients admitted to a tertiary medical ICU between December 2020 and December 2023, who underwent hepatobiliary ultrasonography within six months before admission. MASLD was defined as hepatic steatosis with ≥1 cardiometabolic risk factor. The baseline and 72 h LFTs, injury patterns, and ICU outcomes were compared between MASLD and non-MASLD patients. Logistic regression was used to identify the independent predictors of new-onset LFT elevation and ICU mortality. *Results*: Among 609 patients, MASLD was diagnosed in 240 (39.4%). LFT elevation at admission was more frequent in patients with MASLD (52% vs. 39%, *p* = 0.03), driven mainly by higher alkaline phosphatase (ALP). At 72 h, ALP (96 [67–146] vs. 85 [60–137]) and gamma-glutamyl transferase (GGT) (50 [27–123] vs. 42 [20–100]) levels remained higher in patients with MASLD (*p* < 0.01), although rates of new-onset LFT elevation were similar (*p* > 0.05). Compared to non-MASLD patients, those with MASLD more often required invasive mechanical ventilation (IMV) (64% vs. 33%), central venous catheterization (70% vs. 44%), CRRT (28% vs. 10%), blood product replacement (50% vs. 28%), and developed nosocomial infections (44% vs. 29%) (*p* < 0.05 for all); however, MASLD was not an independent predictor of mortality. The independent risk factors for mortality were IMV, shock, and higher APACHE II scores. *Conclusions*: common among medical ICU patients and is associated with a cholestatic biochemical profile and poor ICU outcomes. However, early hepatic injury and ICU mortality are primarily determined by systemic severity and organ support requirements, not the MASLD itself.

## 1. Introduction

The term “metabolic dysfunction-associated steatotic liver disease (MASLD)” was adopted in 2023 by a Delphi panel including members from 22 related societies, and began to be used instead of the terms “non-alcoholic fatty liver disease (NAFLD)” or “Metabolic Dysfunction-Associated Fatty Liver Disease (MAFLD) [1].” The 2024 Joint European Association for the Study of the Liver (EASL), European Association for the Study of Diabetes (EASD), and European Association for the Study of Obesity (EASO) clinical practice guidelines adopted this terminology and defined MASLD as the presence of hepatic steatosis plus ≥ 1 cardiometabolic risk factors (overweight/obesity, type 2 diabetes, hypertension, dyslipidemia, or insulin resistance) without the need to exclude other liver etiologies [2]. An updated American Association for the Study of Liver Diseases (AASLD) practice guidance issued the same year harmonized the United States recommendations with this nomenclature [3]. The global burden of MASLD is substantial and increasing. Pooled analyses show a prevalence that has increased from 25% in the early 2000s to >30% today [2,4]. Concordantly, MASLD-related mortality has increased rapidly between 2006 and 2023, and is forecast to continue upward through 2040 [4]. MASLD is characterized by low-grade systemic inflammation, mitochondrial dysfunction, and immune metabolic reprogramming, linking it to high vulnerability during acute stress [5].

Elevated liver function tests (LFTs) are detected in up to 20% of critically ill adults and are independently associated with poor outcomes [6]. Several observational cohorts have indicated that steatotic livers are more prone to hypoxic hepatitis, sepsis-associated cholestasis, and drug-induced injury. However, these data are inconsistent, partly because most studies used global intensive care unit (ICU) endpoints (mortality and length of stay) rather than organ-specific outcomes [7]. Experimental work further implicates maladaptive macrophage polarization, oxidative stress, and microvascular dysfunction in sepsis-induced acute liver injury in a MASLD background [8]. Moreover, an LFT-based scoring system that integrates alanine aminotransferase (ALT), aspartate aminotransferase (AST), alkaline phosphatase (ALP), γ-glutamyltransferase (GGT) was shown to stratify patients with MASLD into hepatocellular, mixed, and cholestatic phenotypes, with the cholestatic pattern carrying the worst long-term prognosis [9]. However, their performance in critical care settings remains unexplored.

This study aimed to evaluate the association between pre-existing MASLD and hepatic function during critical illness in non-cirrhotic medical ICU patients. We also examined whether MASLD affects ICU outcomes, particularly mortality, using liver function test changes as an organ-specific indicator of hepatic stress.

## 2. Materials and Methods

### 2.1. Study Design and Setting

This retrospective cohort study was conducted in the tertiary medical ICUs of the Gazi University Hospital with 16 beds. The study included ICU admissions between December 2020 and December 2023. The study protocol was approved by the Local Ethics Committee of Gazi University (Approval Number: 2023-1461) and adhered to the principles of the Declaration of Helsinki.

### 2.2. Patient Selection

**Figure 1:**
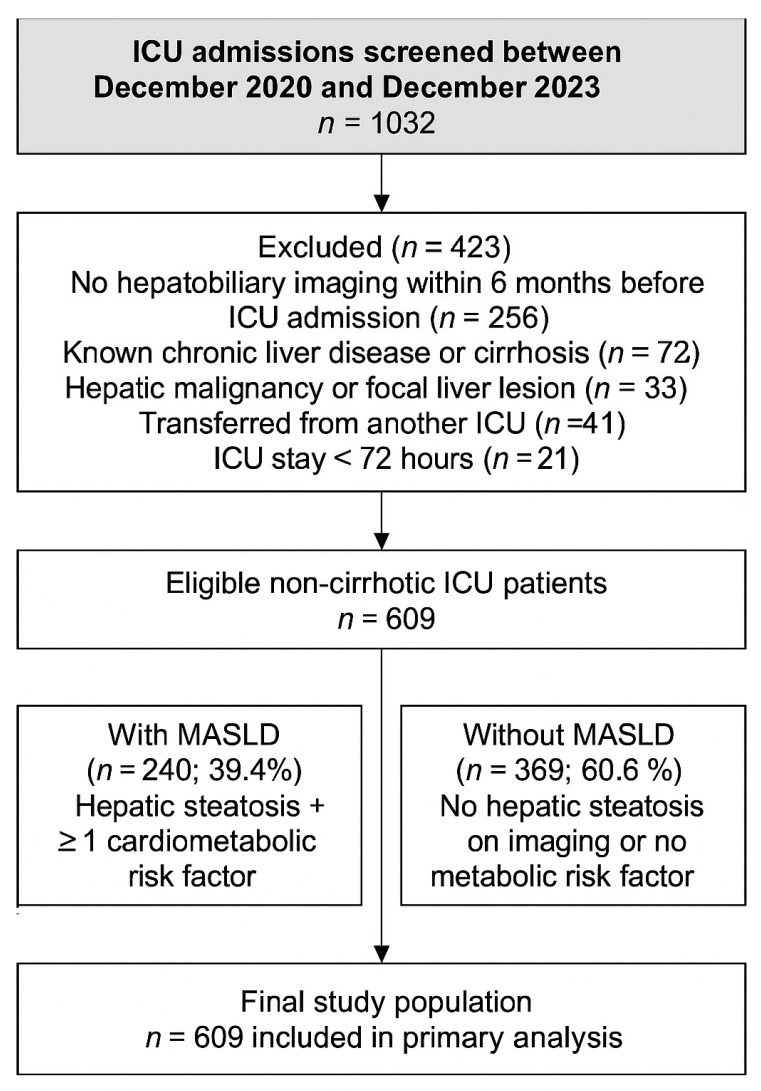
Flowchart showing the selection of medical ICU patients included in the study. A total of 1032 admissions were screened between December 2020 and December 2023. After exclusions for lack of imaging, chronic liver disease, malignancy, transfer, or short ICU stay, 609 non-cirrhotic patients were included and classified according to MASLD status.

Patients aged ≥18 years who underwent hepatobiliary ultrasonographic evaluation at Gazi University Hospital within the past six months before ICU admission were screened for inclusion. A 6-month imaging window was chosen as a conservative definition of pre-existing MASLD. Although current MASLD guidelines recommend less frequent surveillance for non-cirrhotic patients, we used this shorter interval to minimize potential misclassification and to better capture baseline hepatic status prior to critical illness. Patients were excluded if they had no hepatic imaging within the past six months, had a known history of chronic liver disease (e.g., cirrhosis, autoimmune hepatitis, Wilson’s disease), hepatic malignancy, or mass lesions, were transferred from another ICU, or stayed in the ICU for <72 h. A detailed flow diagram of the screening and inclusion process is presented in Figure 1.

### 2.3. Hepatic Steatosis Classification

Hepatic steatosis was classified semiquantitatively based on pre-admission abdominal B-mode ultrasound findings. Grading was performed using established sonographic features, including liver brightness, hepatorenal contrast, visualization of intrahepatic vessels, parenchymal echotexture, and clarity of the diaphragm [10]. According to the conventional classification systems, steatosis severity is defined as follows [10]:

- Grade 0 (absent): normal hepatic echotexture and clear visualization of the portal vein walls and diaphragm.
- Grade 1 (mild): Slight, diffuse increase in liver echogenicity with preserved visualization of the portal vein wall and diaphragm.
- Grade 2 (moderate): Moderate increase in echogenicity with partial obscuration of the portal vein walls and diaphragm.
- Grade 3 (severe): Marked echogenicity with poor or absent visualization of the diaphragm, portal vein walls, and posterior aspect of the right hepatic lobe.

### 2.4. Definition of MASLD

Patients were classified as having MASLD if they fulfilled both of the following criteria in accordance with the 2024 EASL–EASD–EASO Clinical Practice Guideline, the 2023 multisociety Delphi consensus statement, and the 2024 AASLD practice guidance [1,2,3].

#### 2.4.1. Evidence of Hepatic Steatosis

Evidence of hepatic steatosis was based on typical steatotic features on pre-admission abdominal B-mode ultrasound performed within 6 months before ICU admission. Pre-admission abdominal ultrasounds were performed in the radiology department by approximately senior abdominal radiologists and supervised radiology residents under their direct supervision, using standard B-mode criteria for hepatic steatosis. Although current MASLD guidelines accept CAP, MRI-PDFF and histology as alternative diagnostic tools, these modalities were not systematically used in our cohort and were not required for inclusion.

#### 2.4.2. At Least One Cardiometabolic Risk Factor

- Overweight/obesity (BMI ≥ 25 kg m^2^; BMI ≥ 23 kg m^2^ for Asian ancestry).
- Type 2 diabetes mellitus or prediabetes (HbA1c ≥ 5.7% or fasting glucose ≥ 100 mg dL).
- Arterial hypertension (blood pressure ≥ 130/85 mm Hg or antihypertensive therapy).
- Atherogenic dyslipidemia (triglycerides ≥ 150 mg/dL or HDL-C < 40 mg/dL in men and <50 mg/dL in women or lipid-lowering therapy).
- Insulin resistance (HOMA-IR ≥ 2.5) or waist circumference > 94 cm in men and >80 cm in women.

### 2.5. Further Criteria

In addition to the inclusion and exclusion criteria specified under the patient selection subheading, a history of harmful alcohol consumption and other chronic liver diseases was specifically investigated. Individuals who reported data regarding harmful alcohol intake (>210 g week^−1^ in men or >140 g week^−1^ in women) or had another chronic liver disease (viral, autoimmune, cholestatic, genetic, or drug-induced) in their medical files were not classified as having MASLD. Participants with metabolic risk factors who consumed alcohol in the moderate–excessive range (140–350 g week^−1^ in women; 210–420 g week^−1^ in men) were recorded as having metabolic- and alcohol-related liver disease (MetALD) and were excluded from the primary analysis [11].

### 2.6. Data Collection

Demographic data, comorbidities, admission diagnoses, clinical severity scores (Acute Physiology, Age and Chronic Health Evaluation (APACHE II), Sequential Organ Failure Assessment (SOFA)), and ICU interventions (mechanical ventilation, vasopressor use, renal replacement therapy (RRT)) were recorded. Laboratory parameters were collected from electronic health records and patient medical archives. Liver function and related biochemical markers, including ALT, AST, ALP, GGT, total and direct bilirubin, prothrombin time (PT), activated partial thromboplastin time (aPTT), international normalized ratio (INR), albumin, and creatinine, were recorded at ICU admission and on the third day of ICU stay (72 h time point).

Nutritional support followed a unit protocol with early enteral feeding whenever feasible; due to this standardized approach and its close relation to overall illness severity, detailed nutritional variables were not included as primary predictors in multivariable models.

### 2.7. Definition of Elevated Liver Function Tests

#### 2.7.1. Time Points and Dynamic Assessment

Liver function tests (LFTs)—ALT, AST, ALP, GGT, and total/direct bilirubin levels—were obtained at hospital admission (baseline, *t*0, within the first 6 h) and 72 ± 6 h (*t*72). Follow-up LFTs at 72 ± 6 h (t72) were chosen to provide a uniform assessment of early hepatic injury. This time point is routinely available in our ICU and aligns with prior studies evaluating early hepatic dysfunction in critically ill patients. Beyond 72 h, laboratory sampling was not systematic and was therefore not included in the primary analyses.

ALT, AST, ALP, GGT and total/direct bilirubin were measured using standard assays; abnormal results were defined as values above the laboratory-specific upper limit of normal (ULN).

#### 2.7.2. Definition of “Abnormal”

For each analyte, the values were interpreted as multiples of assay-specific ULN. Any value > the ULN was considered abnormal. To ensure a hepatic origin for cholestatic markers, elevated ALP required a concomitant elevation of GGT (≥ULN) when available. Hyperbilirubinemia was defined as total bilirubin > ULN; when fractionation was available, direct (conjugated) bilirubin > ULN or >50% of total indicated a cholestatic component. Albumin and INR were recorded as indices of synthetic function but were not used for pattern classification.

#### 2.7.3. Pattern Classification (Primary Analysis)

The pattern of liver injury at each time point was classified using the conventional R-ratio, defined as:

$$R=(ALT/ULN_{ALT})/(ALP/ULN_{ALP}).$$

When both ALT and AST were available, the higher aminotransferase value was used.

Hepatocellular pattern: R≥5; cholestatic pattern: R≤2; mixed pattern: 2<R<5.

#### 2.7.4. Sensitivity (Alternative Pragmatic Rule, Pre-Specified)

For robustness, we repeated the analysis using a pragmatic predominance rule: hepatocellular pattern defined as ALT or AST ≥ 3 × ULN with ALP < 2 × ULN; cholestatic pattern as ALP ≥ 2 × ULN and/or direct bilirubin > ULN; mixed pattern when both criteria were present. The simplified classification yielded results consistent with the R-ratio-based analysis unless otherwise specified.

### 2.8. Outcome Measures and Grouping

After applying the exclusion criteria, all eligible patients admitted to the ICU were included in the primary cohort. LFT values on ICU admission were used to classify patients as having normal or elevated liver biochemistry and subtyping LFT abnormalities. To evaluate the association between MASLD and new-onset LFT elevation during ICU stay, a predefined subgroup analysis was performed in patients whose LFTs were within the normal limits at the time of ICU admission. In this subgroup, we assessed the incidence of LFT elevation at 72 h and its relationship with underlying MASLD status. The primary outcome was the presence of baseline and new-onset LFT Elevation in critically ill patients during the ICU stay. Secondary analyses included comparisons of standard ICU outcomes, including mortality, length of stay, need for mechanical ventilation, vasopressor use, and renal replacement therapy between patients with and without steatosis. For further comparative analysis, the patients were stratified based on ICU survival status (survivors vs. non-survivors).

### 2.9. Statistical Analysis

Descriptive statistics were reported as mean ± standard deviation or median with interquartile range (IQR) for continuous variables and frequency (percentage) for categorical variables. Patients were compared across groups based on their steatosis status and ICU survival. The chi-square or Fisher’s exact test was used for categorical comparisons, and the Mann–Whitney U or Student’s *t*-test was used for continuous variables, depending on the distribution. Logistic regression analyses were conducted to identify independent predictors of LFT elevation and ICU mortality. Statistical significance was set at *p* < 0.05. All analyses were performed using SPSS Version 22.0 (IBM Corp., Armonk, NY, USA).

## 3. Results

We included 609 non-cirrhotic patients in the medical ICU. The baseline characteristics of the ICU patients according to the presence of MASLD are shown in Table 1. MASLD was diagnosed in 240 (39.4%) of all patients. Shock at admission was more frequent in MASLD (38% vs. 14%; *p* < 0.01). Baseline laboratory tests showed higher ALP in the MASLD group (median 103 vs. 89 IU/L, *p* = 0.03), with other LFTs similar. Overall, 282/609 (46%) patients had LFT elevation at ICU admission, which was more frequent in MASLD (52% vs. 39%, *p* = 0.03). The patterns of injury at presentation (hepatocellular, cholestatic, mixed) were not statistically different between the groups (*p* > 0.05).

Follow-up data regarding 72nd-hour laboratory tests, supportive therapy, and clinical outcomes according to the presence of MASLD are given in Table 2. By hour 72, ALP and GGT remained higher in the MASLD group (ALP 96 vs. 85 IU/L, *p* = 0.01; GGT 50 vs. 42 IU/L, *p* = 0.02), whereas ALT, AST, and bilirubin levels were similar (*p* > 0.05). Any LFT elevation at hour-72 occurred in 333/609 (55%) patients, trending higher in patients with MASLD (57% vs. 49%, *p* = 0.06). In the predefined subgroup with normal baseline LFTs (*n* = 327), new-onset LFT elevation at 72 h occurred at similar frequencies in patients with MASLD and without MASLD (16% vs. 16%, *p* = 0.25). Patients with MASLD required Invasive Mechanical Ventilation (IMV) more often (64% vs. 33%, *p* < 0.01) and underwent central venous catheterization more frequently (70% vs. 44%, *p* < 0.01). Additionally, in these patients, nosocomial infections were more common (44% vs. 29%, *p* < 0.01), as was use of continuous renal replacement therapy (CRRT 28% vs. 10%, *p* < 0.01) and blood product replacement (50% vs. 28%, *p* < 0.01), than in patients without MASLD. Notably, shock at ICU admission was more frequent in patients with MASLD compared with those without MASLD (38% vs. 14%), indicating greater initial hemodynamic instability in the MASLD group.

ICU length of stay was modestly longer (median 7 [IQR 4–17] vs. 6 [4–12] days, *p* = 0.04), and ICU mortality was substantially higher in patients with MASLD vs. without MASLD (61% vs. 17%, *p* < 0.01).

Factors related to new-onset LFT elevation are shown in Table 3. In the predefined subgroup of 327 patients with normal baseline LFTs, univariate analysis showed that a higher admission SOFA score, need for IMV, and presence of shock were associated with new-onset LFT elevation at 72 h. For new-onset LFT elevation in patients with normal baseline LFTs, only higher admission SOFA independently increased risk (OR 95% CI: 1.09 [1.03–1.17], *p* < 0.01) (Table 4).

Independent predictors of ICU mortality were IMV (OR 95% CI: 21.6 [9.7–48.7], *p* < 0.01), shock (OR 95% CI: 4.3 [2.2–8.4], *p* < 0.01), and higher APACHE-II (OR 95% CI: 1.06 [1.02–1.12], *p* = 0.01). MASLD status was not defined as an independent predictor (Table 4).

## 4. Discussion

In this single-center medical ICU cohort, pre-admission MASLD was associated with more frequent LFT elevation and higher ALP on ICU admission, higher ALP and GGT at 72nd hour, and worse ICU outcomes, including ICU mortality, need for organ support therapies, and higher ICU length of stay.

However, excess mortality was explained by illness severity and organ support requirements, whereas MASLD itself was not independently associated with new-onset LFT elevation or death. Our findings suggest that pre-existing MASLD primarily modifies the biochemical pattern of liver stress in critical illness, biasing toward a cholestatic response with higher ALP at admission and persistently higher ALP/GGT at 72 h, rather than increasing the incidence of early hepatic injury or directly driving mortality. Among patients with normal baseline LFTs, new-onset LFT elevation at 72 h was determined by systemic severity, not MASLD status, and ICU mortality was explained by IMV, shock, and APACHE-II, with no independent effect of MASLD. Mechanistically, MASLD may confer cholangiocyte/bile acid transporter vulnerability under inflammatory and hypoperfusion stress, yielding a cholestatic-predominant profile without necessarily causing higher transaminase release. In a heterogeneous ICU population, these MASLD-related susceptibilities are overridden by acute illness intensity; therefore, MASLD acts as a phenotype-shaping risk marker rather than a stand-alone prognostic driver, and an independent effect may be more evident in sepsis- or shock-specific cohorts.

MASLD is now the globally endorsed term for fatty liver linked to cardiometabolic dysfunction, and current statements underscore its scale and the need to consider it across care settings, including critical illness [1,2].

In our cohort, 46% of the patients had abnormal LFTs at admission, and ALP was higher in patients with MASLD with persistently higher ALP/GGT at 72 h. This aligns with previous ICU data showing that acquired liver dysfunction during ICU stay is common and often cholestasis is predominantly driven by inflammation-mediated transporter downregulation, hypoperfusion, and sepsis-related canalicular dysfunction [12,13]. Reviews have reported secondary acquired cholestasis in a substantial fraction of ICU patients, with bilirubin and cholestatic enzymes frequently elevated early in the course.

In our cohort, shock at ICU admission was more frequent among patients with MASLD, indicating a sicker baseline profile and contributing to the markedly worse crude outcomes observed in this group. Given that MASLD is increasingly recognized as a systemic pro-inflammatory and cardiometabolic condition, it is biologically plausible that MASLD-related endothelial dysfunction, atherosclerosis, and cardiac/metabolic comorbidities may reduce hemodynamic reserve and predispose these patients to hemodynamic instability during critical illness. However, our retrospective single-center design and limited sample size do not allow us to determine whether MASLD itself is an independent causal risk factor for shock. This association should therefore be considered hypothesis-generating and warrants confirmation in prospective studies. After adjustment for shock and global illness severity, MASLD was not independently associated with early liver injury or ICU mortality, suggesting that systemic severity rather than hepatic steatosis per se remains the main driver of prognosis in this setting. Previous critical care studies have consistently shown that hypotension and vasopressor requirement are among the strongest predictors of death regardless of pre-existing comorbidities [14,15]. Future studies with larger, homogeneous cohorts are required to clarify whether patients with MASLD have a true predisposition to circulatory collapse in critical illness or whether the observation in our study was incidental.

At baseline, patients with MASLD had significantly higher ALP levels, whereas other liver enzymes (ALT, AST, and bilirubin) were not elevated. One could expect broader abnormalities in MASLD since the disease involves hepatic steatosis and varying degrees of ongoing hepatocellular stress. However, this was not the case in our cohort. Patients with MASLD had higher ALP and GGT levels at admission, indicating a tendency toward a more cholestatic biochemical profile, although the proportion of patients meeting the predefined cholestatic injury pattern did not differ significantly between groups. A potential explanation is that ALP elevation in MASLD may reflect cholestatic or metabolic stress rather than direct hepatocellular injury. Another possibility is that the higher shock frequency in the MASLD group contributed indirectly to cholestatic enzyme elevation through early hypoperfusion and microcirculatory dysfunction, while transaminases remained within similar ranges between groups. This interpretation aligns with reports that critically ill cholestasis can develop rapidly under hemodynamic instability and systemic inflammation, whereas transaminase elevation usually requires more severe or sustained hepatocellular damage [15,16].

Interestingly, the distribution of liver injury patterns (hepatocellular, cholestatic, mixed) at admission did not significantly differ between the groups with or without MASLD. This contrasts with our initial hypothesis and prior literature highlighting a predominance of cholestatic abnormalities in critically ill patients, especially those with underlying steatotic liver disease. There are several possible explanations for this finding. First, the classification of hepatocellular versus cholestatic patterns may not be sufficiently sensitive to capture subtle shifts in enzyme profiles. Second, the early phase of critical illness is characterized by multiple overlapping insults, including hypoxia, inflammation, and drug exposure, such that the contribution of MASLD may be obscured. Prior reviews of ICU-associated liver dysfunction have emphasized that while cholestasis is common overall, its occurrence is not restricted to patients with metabolic liver disease but is instead a shared manifestation of systemic stress. Therefore, the absence of a clear difference in injury patterns in our data is consistent with the notion that acute critical illness overwhelms disease-specific patterns and produces various biochemical phenotypes across patient groups [12,17]. The higher prevalence of shock at admission among MASLD patients reflects a sicker baseline profile and may partly explain their worse crude ICU outcomes. After adjustment for shock and global illness severity, MASLD itself was not associated with early liver injury or ICU mortality, suggesting that systemic severity rather than hepatic steatosis per se drives prognosis in this setting.

Among patients with normal baseline LFTs, new-onset elevation at 72 h did not differ by MASLD status; in multivariable analysis, only higher admission SOFA scores independently predicted this outcome. This supports the concept that early hepatic injury in the ICU primarily reflects systemic severity (hypotension, tissue hypoxemia, and inflammation) rather than pre-existing steatosis. Mechanistic work in sepsis demonstrates that cytokine-mediated bile acid transporter impairment, microcirculatory dysfunction, and mitochondrial injury are proximate drivers of acute hepatic dysfunction phenomena that are tightly linked to higher organ failure scores [13,16].

Patients with MASLD more often required IMV and CRRT, developed nosocomial infections, and had higher ICU mortality. However, MASLD was not an independent predictor of death. Prior data are mixed; in a sepsis-only cohort (SepsisFAT), steatotic liver disease independently increased 30-day mortality, whereas retrospective ICU studies found no excess risk after adjusting for severity [18,19].

Our data fit the contemporary pathobiological view that MASLD is a systemic, immunometabolic disorder predisposing the liver—especially cholangiocytes and bile acid transport—to stress-induced dysfunction rather than to frank hepatocellular necrosis in the early ICU window. This framework helps explain why a cholestatic-predominant biochemical response (ALP/GGT) was more evident than transaminase release in patients with MASLD, while early hepatic injury incidence remained driven by global severity (SOFA) and sentinel events (shock, IMV) [20].

Inflammation-insulin resistance crosstalk and altered innate immunity in MASLD may amplify cholestatic responses to sepsis, hypoperfusion, and parenteral/enteral nutrition—typical ICU exposures—without necessarily increasing hepatocellular injury rates at 72 h. This aligns with evidence that MASLD’s inflammatory tone (NF-κB–centric signaling; cytokine-lipotoxicity loops) heightens vulnerability to secondary hits [21].

Mechanistically, TNF–receptor pathway adaptors (TRADD/TRAF axis) regulate NF-κB and cell-death checkpoints in hepatocytes and cholangiocytes; stress-context switching at this node is a biologically plausible substrate for the cholestatic phenotype we observed [22]. In parallel, sepsis-related innate-immune activation and endothelial injury further perturb bile acid transport and microcirculatory flow, favoring cholestasis in the critically ill [23].

Our results align with the latter and indicate that in a heterogeneous ICU population, acute severity and organ support needs dominate prognosis and may overshadow any direct effect of MASLD. The persistently higher ALP/GGT ratio in MASLD points to a cholestatic response to systemic stress, but this pattern alone did not translate into additional early hepatic injury or mortality once severity was accounted for. Prospective phenotype-specific studies are warranted to determine when MASLD exerts an independent prognostic impact.

According to the results of the current study, MASLD appears to mark patients at a higher risk for cholestatic LFT abnormalities during critical illness, calling for early avoidance of cholestatic/hepatotoxic exposures where alternatives exist, in addition to aggressive hemodynamic optimization and infection control. However, predicting new-onset LFT elevation or mortality should rely on severity measures (SOFA/APACHE-II) and sentinel events (shock, IMV), not MASLD status alone, in a general ICU.

Limitations of the current study include the retrospective single-center design, misclassification risk, limited fibrosis staging due to steatosis ascertainment by ultrasound within six months, lack of exposure and response data for potentially hepatotoxic drugs, and the possibility of residual confounding by unmeasured illness severity factors. External validation in prospective ICU cohorts with standardized imaging and adjudicated drug exposure is required.

We did not have systematic CAP, MRI-PDFF, FibroScan or histology to validate ultrasound-based steatosis, which may lead to misclassification; however, conventional ultrasound is an accepted first-line tool for detecting moderate-to-severe steatosis and is widely used in epidemiologic MASLD cohorts.

Medication exposure data (e.g., acetaminophen, antibiotics) were not uniformly recorded in electronic files and thus were excluded from multivariable models; this remains a limitation.

Another potential limitation is that hepatic steatosis was diagnosed using conventional ultrasound performed by operators with varying seniority and experience, raising concerns about interobserver variability in a semi-quantitative technique. Although this represents a degree of subjectivity, previous reports have shown acceptable reproducibility of ultrasound-based steatosis assessment, even between radiologists with different levels of expertise [20]. Therefore, while operator dependence should be acknowledged, we consider that this issue does not constitute a major threat to the validity of our findings, as our results are consistent with prior ICU and MASLD cohorts, where ultrasound was used for case ascertainment.

## 5. Conclusions

MASLD is common among medical ICU patients and is associated with cholestatic elevation of the LFT profile and worse ICU outcomes; however, early hepatic injury and mortality are primarily driven by systemic severity and organ support needs. Future work should test hepatoprotective strategies in ICU patients with MASLD, while integrating severity-based risk tools.

## Figures and Tables

**Table 1:** Baseline characteristics of ICU patients according to the presence of MASLD.

Characteristics	All Patients *n* = 609	MASLD (+) *n* = 240 (39.4%)	MASLD (−) *n* = 369 (60.6%)	*p* Value
Age (years)	73 (64–81)	73 (64–81)	73 (63–81)	0.69
Female, *n* (%)	273(45%)	97(40%)	176(44%)	0.04
Admission source, *n* (%) Emergency Department Inpatient Wards	343 (56%) 266 (44%)	110 (46%) 116 (48%)	233 (59%) 150 (38%)	0.03
Comorbidities, *n* (%) Chronic renal disease Pulmonary disease Hypertension Neurological Rheumatological Diabetes Mellitus Malignancies	200 (33%) 222 (36%) 351 (58%) 130 (21%) 40 (7%) 218 (36%) 183 (30%)	82 (34%) 94 (39%) 150 (63%) 43 (18%) 18 (8%) 92 (38%) 84 (35%)	118 (30%) 128 (32%) 201 (51%) 87 (22%) 22 (6%) 126 (32%) 99 (25%)	0.33 0.11 0.12 0.05 0.18 0.44 0.28
BMI (kg/m2)	24.2 (22.5–28.2)	24.6 (22.5–28.9)	24.2 (22.5–27.8)	0.46
APACHE-II Score	22 (16–29)	22 (16–30)	21 (16–29)	0.06
SOFA Score	6 (3–9)	6 (3–10)	5 (3–8)	0.16
Glasgow Coma Scale	14 (10–15)	14 (10–15]	15 (10–15)	0.11
RIFLE stage, *n* (%) Risk Injury Failure Loss ESRD	125 (21%) 88 (14%) 117 (19%) 26 (4%) 62 (10%)	47 (20%) 39 (16%) 48 (20%) 11 (5%) 31 (13%)	78 (20%) 49 (12%) 69 (17%) 15 (4%) 31 (8%)	0.36 0.18 0.38 0.45 0.04
Reason of ICU Admission, *n* (%) Respiratory failure Sepsis Renal failure Cardiac decompensation Acute neurological disorders Metabolic disturbances Surgery	346 (57%) 339 (56%) 273 (45%) 95 (16%) 68 (11%) 31 (5%) 24 (4%)	147 (61%) 108 (45%) 141 (59%) 42 (18%) 26 (11%) 11 (5%) 9 (4%)	192 (48%) 65 (16%) 205 (52%) 53 (13%) 42 (11%) 20 (5%) 15 (4%)	0.06 0.07 0.28 0.18 0.47 0.39 0.51
AKI at ICU Admission, *n* (%)	345 (57%)	141 (59%)	204 (52%)	0.06
Shock at ICU Admission, *n* (%)	144 (24%)	90 (38%)	54 (14%)	<0.01
Laboratory Tests on ICU Admission ALT (>35 IU/L) AST (>35 IU/L) ALP (>104 IU/L) GGT (>42 IU/L) TB (>1.2 mg/dL) DB (>0.3 mg/dL) Albumin (<3.5 g/dL) Creatinine (>0.9 mg/dL) PT (>14.5 s) aPTT (aPTT > 32 s) INR (INR > 1.2)	24 (14–57) 33 (21–71) 93 (67–144) 43 (29–100) 0.78 (0.48–1.31) 0.29 (0.16–0.62) 2.9 ± 0.6 1.51 (0.89–2.49) 14.6 (13–18) 29 (24.6–32.2) 1.24 (1.11–1.49)	27 (14–61) 33 (22–65) 103 (69–148) 46 [26–118) 0.8 (0.48–1.47) 0.3 (0.15–0.69) 2.9 ± 0.7 1.43 (0.9–3.26) 14.6 (13–18.4) 29.6 (24.7–36.9)] 1.24 (1.09–1.55)	23 (13–53) 32 (20–74) 89 (65–139) 40 (22–95) 0.74 (0.47–1.22) 0.28 (0.16–0.60) 2.9 ± 0.6 1.52 (0.89–2.81) 14.6 (13–17.4) 28.8 (23.6–33.2) 1.24 (1.11–1.50)	0.91 0.97 0.03 0.11 0.23 0.68 0.19 0.08 0.21 0.26 0.06
LFT Elevation on ICU Admission Hepatocellular injury Cholestatic pattern Mixed injury	282 (46%) 101 (17%) 81 (13%) 100 (16%)	124 (52%) 45 (19%) 35 (15%) 44 (18%)	158 (39%) 56 (14%) 46 (12%) 56 (14%)	0.03 0.11 0.26 0.18

Values are presented as mean ± SD, median [IQR], or *n* (%). MASLD: Metabolic dysfunction-associated steatotic liver disease. ICU: Intensive Care Unit; APACHE-II: Acute Physiology and Chronic Health Evaluation II; SOFA: Sequential Organ Failure Assessment. ESRD, end-stage renal disease; ALT, alanine aminotransferase; AST, aspartate aminotransferase; ALP, alkaline phosphatase; GGT, gamma-glutamyl transferase; TB, total bilirubin; DB, direct bilirubin; PT, prothrombin time; aPTT, activated partial thromboplastin time; INR, international normalized ratio; LFT, liver function test.

**Table 2:** ICU follow-up data regarding 72nd-hour laboratory tests, supportive therapy, and clinical outcomes according to the presence of MASLD.

Characteristics	All Patients *n* = 609	MASLD (+) *n* = 240(39.4%)	MASLD (-) *n* = 369(60.6%)	*p* Value
Laboratory Tests on 72nd Hour ALT (>35 IU/L) AST (>35 IU/L) ALP (>104 IU/L) GGT (>42 IU/L) TB (>1.2 mg/dL) DB (>0.3 mg/dL)	26 (15–52) 31 (19–62) 87 (63–138) 44 (22–106) 0.69 (0.46–1.25) 0.27 (0.15–0.56)	27 (16–48) 34 (21–68) 96 (67–146) 50 (27–123) 0.69 (0.49–1.31) 0.28 (0.16–0.6)	25 (14–53) 28 (18–56) 85 (60–137) 42 (20–100) 0.69 (0.45–1.21) 0.27 (0.14–0.56)	0.65 0.25 0.01 0.02 0.56 0.33
LFT Elevation on 72nd Hour Hepatocellular injury Cholestatic pattern Mixed injury	333 (55%) 153 (25%) 93 (15%) 87 (14%)	137 (57%) 67 (28%) 35 (15%) 35 (15%)	196 (49%) 86 (22%) 58 (15%) 52 (13%)	0.06 0.18 0.39 0.47
New-Onset LFT Elevation on 72nd Hour (*n* = 327 Patients with no LFT Elevation on ICU Admission)	101 (17%)	39 (16%)	62 (16%)	0.25
Parenteral or Enteral Nutrition, *n* (%)	510 (88%)	202 (84%)	308 (78%)	0.42
Requirement of respiratory support, *n* (%) Invasive mechanical ventilation Non-invasive mechanical ventilation High-flow nasal oxygen therapy	284 (47%) 145 (24%) 55 (10%)	153 (64%) 44 (18%) 18 (8%)	131 (33%) 101 (26%) 37 (9%)	<0.01 <0.01 0.16
Development of ARDS, *n* (%)	24 (4%)	14 (6%)	10 (3%)	0.12
Central Venous Catheterization, *n* (%)	343 (56%)	169 (70%)	174 (44%)	<0.01
Nosocomial Infection	219 (36%)	105 (44%)	114 (29%)	<0.01
Requirement of RRT, *n* (%) Hemodialysis Continuous renal replacement therapy	158 (26%) 107 (18%)	72 (30%) 68 (28%)	86 (22%) 39 (10%)	0.06 <0.01
Blood Product Replacement, *n* (%)	229 (38%)	119 (50%)	110 (28%)	<0.01
ICU Mortality	216 (35%)	147 (61%)	69 (17%)	<0.01
Length of ICU Stay	6 (4–13)	7 (4–17)	6 (4–12)	0.04

Values are presented as mean ± SD, median [IQR], or *n* (%). MASLD: Metabolic dysfunction-associated steatotic liver disease. LFT, liver function test. ICU: Intensive Care Unit; ALT, alanine aminotransferase; AST, aspartate aminotransferase; ALP, alkaline phosphatase; GGT, gamma-glutamyl transferase; TB, total bilirubin; DB, direct bilirubin.

**Table 3:** Factors in relation to new-onset LFT elevation.

Characteristics	All Patients *n* = 327	New Onset Elevation (+) *n* = 101 (31%)	New Onset Elevation (-) *n* = 226 (69%)	*p* Value
Age (years)	73 (66–81)	72 (66–81)	74 (65–82)	0.15
Female, *n* (%)	149 (47%)	42 (60%)	107 (47%)	0.2
Admission source, *n* (%) Emergency Department Inpatient Wards	185 (57%) 142 (43%)	55 (79%) 46 (66%)	130 (58%) 96 (42%)	0.32 0.42
Comorbidities, *n* (%) Hypertension Pulmonary disease Diabetes Mellitus Chronic renal disease Malignancies Neurological Rheumatological	199 (44%) 135 (41%) 117 (36%) 111 (34%) 93 (28%) 84 (26%) 21 (6%)	61 (87%) 41 (59%) 35 (50%) 36 (51%) 24 (34%) 33 (47%) 9 (13%)	138 (61%) 94 (42%) 82 (36%) 75 (33%) 69 (31%) 51 (23%) 12 (5%)	0.17 0.04 0.42 0.48 0.32 0.26 0.18
BMI (kg/m^2^)		24.8 (23.5–29.4)	24.3 (22.5–28)	0.45
APACHE-II Score	20 (15–27)	22 (15–27)	20 (14–27)	0.26
SOFA Score	5 (3–8)	6 (4–10)	4 (2–8)	<0.01
Glasgow Coma Scale	15 (12–15)	14 (10–15)	15 (12–15)	<0.01
RIFLE stage, *n* (%) Risk Injury Failure Loss ESRD	73 (22%) 43 (13%) 53 (16%) 13 (4%) 30 (9%)	17 (24%) 15 (21%) 15 (21%) 8 (11%) 9 (13%)	56 (25%) 28 (12%) 38 (17%) 5 (2%) 21 (9%)	0.07 0.33 0.38 0.02 0.55
Reason for ICU Admission, *n* (%) Respiratory failure Sepsis Renal failure Cardiac decompensation Acute neurological disorders Metabolic disturbances Surgery	187 (57%) 153 (47%) 135 (41%) 47 (14%) 43 (13%) 13 (4%) 9 (3%)	52 (74%) 55 (79%) 44 (63%) 14 (20%) 19 (27%) 6 (9%) 4 (6%)	135 (60%) 98 (43%) 89 (39%) 33 (15%) 24 (11%) 7 (3%) 5 (2%)	0.09 0.02 0.04 0.49 0.03 0.18 0.29
AKI at ICU Admission, *n* (%)	178 (54%)	53 (76%)	125 (55%)	0.35
Shock at ICU Admission, *n* (%)	67 (20%)	23 (33%)	44 (19%)	0.01
Requirement of mechanical ventilation, *n* (%)				
Invasive mechanical ventilation	126 (39%)	54 (54%)	72 (32%)	0.01
Non-invasive mechanical ventilation	91 (28%)	23 (23%)	68 (30%)	0.18
Presence of MASLD	116 (35%)	39 (56%)	77 (34%)	0.25
Hepatic Steatosis Grade Grade 1 Grade 2 Grade 3	69 (21%) 39 (12%) 20 (6%)	24 (34%) 12 (17%) 5 (7%)	45 (20%) 27 (12%) 15 (7%)	0.18 0.39 0.47
ICU Mortality	94 (29%)	40 (57%)	54 (24%)	<0.01

Values are presented as mean ± SD, median [IQR], or *n* (%). ICU: Intensive Care Unit; APACHE-II: Acute Physiology and Chronic Health Evaluation II; SOFA: Sequential Organ Failure Assessment. ESRD, end-stage renal disease; LFT, liver function test; BMI: Body mass index; AKI: Acute Kidney Injury.

**Table 4:** Independent risk factors for ICU mortality and new-onset LFT elevation.

	Adjusted OR [95% CI]	*p* Value
Mortality (*n* = 609)		
Invasive Mechanical Ventilation	21.6 [9.7–48.7]	<0.01
Shock	4.3 [2.2–8.4]	<0.01
APACHE-II Score	1.06 [1.02–1.12]	0.01
New-Onset LFT Elevation (*n* = 327)		
SOFA Score on ICU Admission	1.09 [1.03–1.17]	<0.01

*n* = number, ICU: Intensive care unit; APACHE-II: Acute Physiology and Chronic Health Evaluation II; LFT, liver function test; SOFA: Sequential Organ Failure Assessment.

## Data Availability

The data presented in this study are available upon request from the corresponding author.
